# Measurement of Human Walking Movements by Using a Mobile Health App: Motion Sensor Data Analysis

**DOI:** 10.2196/24194

**Published:** 2021-03-05

**Authors:** Sungchul Lee, Ryan M Walker, Yoohwan Kim, Hyunhwa Lee

**Affiliations:** 1 School of Computing and Information Systems Grand Valley State University Allendale, MI United States; 2 Computer Science University of Nevada, Las Vegas Las Vegas, NV United States; 3 School of Nursing University of Nevada, Las Vegas Las Vegas, NV United States

**Keywords:** mobile health, mHealth, walking balance, smartphone, motion sensor, sensor, walking, walking balance, mobile phone

## Abstract

**Background:**

This study presents a new approach to measure and analyze the walking balance of humans by collecting motion sensor data in a smartphone.

**Objective:**

We aimed to develop a mobile health (mHealth) app that can measure the walking movements of human individuals and analyze the differences in the walking movements of different individuals based on their health conditions. A smartphone’s motion sensors were used to measure the walking movements and analyze the rotation matrix data by calculating the variation of each xyz rotation, which shows the variables in walking-related movement data over time.

**Methods:**

Data were collected from 3 participants, that is, 2 healthy individuals (1 female and 1 male) and 1 male with back pain. The participant with back pain injured his back during strenuous exercise but he did not have any issues in walking. The participants wore the smartphone in the middle of their waistline (as the center of gravity) while walking. They were instructed to walk straight at their own pace in an indoor hallway of a building. The walked a distance of approximately 400 feet. They walked for 2-3 minutes in a straight line and then returned to the starting location. A rotation vector in the smartphone, calculated by the rotation matrix, was used to measure the pitch, roll, and yaw angles of the human body while walking. Each xyz-rotation vector datum was recalculated to find the variation in each participant’s walking movement.

**Results:**

The male participant with back pain showed a diminished level of walking balance with a wider range of xyz-axis variations in the rotations compared to those of the healthy participants. The standard deviation in the xyz-axis of the male participant with back pain was larger than that of the healthy male participant. Moreover, the participant with back pain had the widest combined range of right-to-left and forward-to-backward motions. The healthy male participant showed smaller standard deviation while walking than the male participant with back pain and the female healthy participant, indicating that the healthy male participant had a well-balanced walking movement. The walking movement of the female healthy participant showed symmetry in the left-to-right (x-axis) and up-to-down (y-axis) motions in the x-y variations of rotation vectors, indicating that she had lesser bias in gait than the others.

**Conclusions:**

This study shows that our mHealth app based on smartphone sensors and rotation vectors can measure the variations in the walking movements of different individuals. Further studies are needed to measure and compare walking movements by age, gender, as well as types of health problems or disease. This app can help in finding differences in gait in people with diseases that affect gait.

## Introduction

Human balance is achieved and maintained by a complex set of human sensorimotor and musculoskeletal systems that control vision, proprioception, vestibular function, muscle contraction, and others. Multiple factors such as psychological factors, injury, or disease can affect these components [1]. Postural balance is commonly used for measurement in healthy and pathological participants and is used for diagnosing disorders related to the nervous system [2], such as ataxia [3], cognitive deficits [4-6], Parkinson disease [7,8], vision problems [9], Alzheimer [4], and so on. Walking balance is also a good method for measuring human postural balance, as it requires the coordinated use of the visual, vestibular, and musculoskeletal systems [10]. Walking balance can become less stable and can fluctuate if an individual has experienced a stroke [11] or a lower limb or back injury [12] due to fragile biomechanical structures in the sensorimotor and musculoskeletal systems that influence how the human body moves while walking [13,14].

Mobile health (mHealth) supports methods that measure physical activities [15] such as measuring human balance and stability by using gravity, linear acceleration, and orientation [16,17]. Moreover, in previous mHealth research, smartphones have been used to support the diagnosis of diseases related to human balance, such as Parkinson disease [18]. However, those studies did not fully utilize smartphone sensors even though smartphones have multiple physical and software sensors. Those studies have also not considered the previous steps even though human steps affect the next step. Additionally, the x, y, and z axes of the motion sensors should be analyzed to obtain a detailed understanding of the human walking movement.

Our study introduces a new method to measure the walking balance by using motion sensors in a smartphone to determine the rotation vector. The rotation vector provides the pitch, roll, and yaw angles in the smartphone. We can, therefore, measure the pitch, roll, and yaw angles of the human body while walking by using the smartphone worn around the body waistline. Data were collected from 1 healthy female, 1 healthy male, and 1 male with back pain wearing the smartphone while walking. The male experiencing back pain injured his back during exercise but he did not have any issues walking. His back pain was confirmed by his doctor’s note.

We developed an mHealth app to record and analyze the sensor output in the smartphone while participants walk. Pitch, roll, and yaw angles were extrapolated from the recorded sensor data, and a model was created to compare the differences in the walking balance among the participants. We used the ggplot2 graphics package in R programming language to create the visualizations.

## Methods

### Implementation of the mHealth App

**Figure 1:**
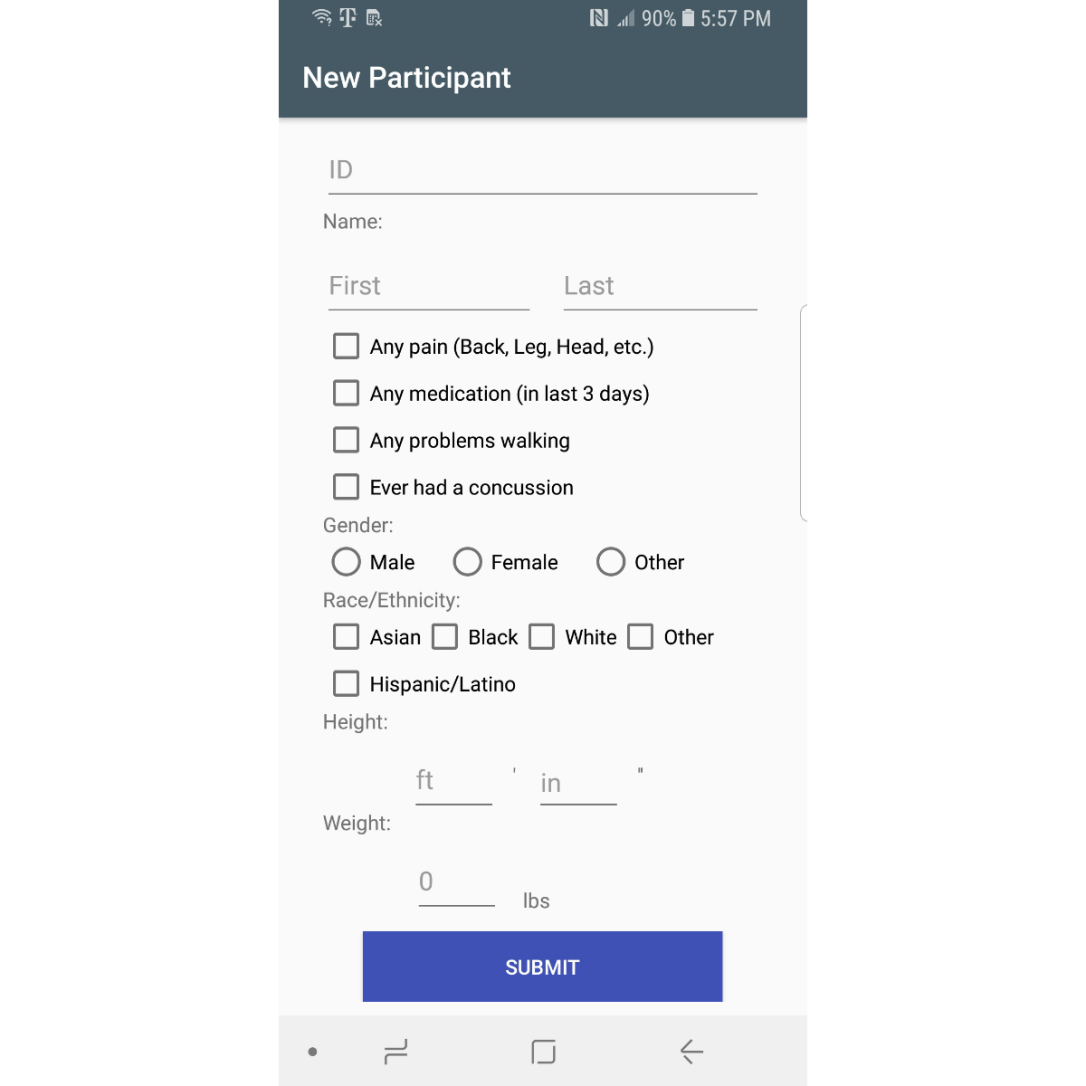
Main fragment of the mobile health app.

**Figure 2:**
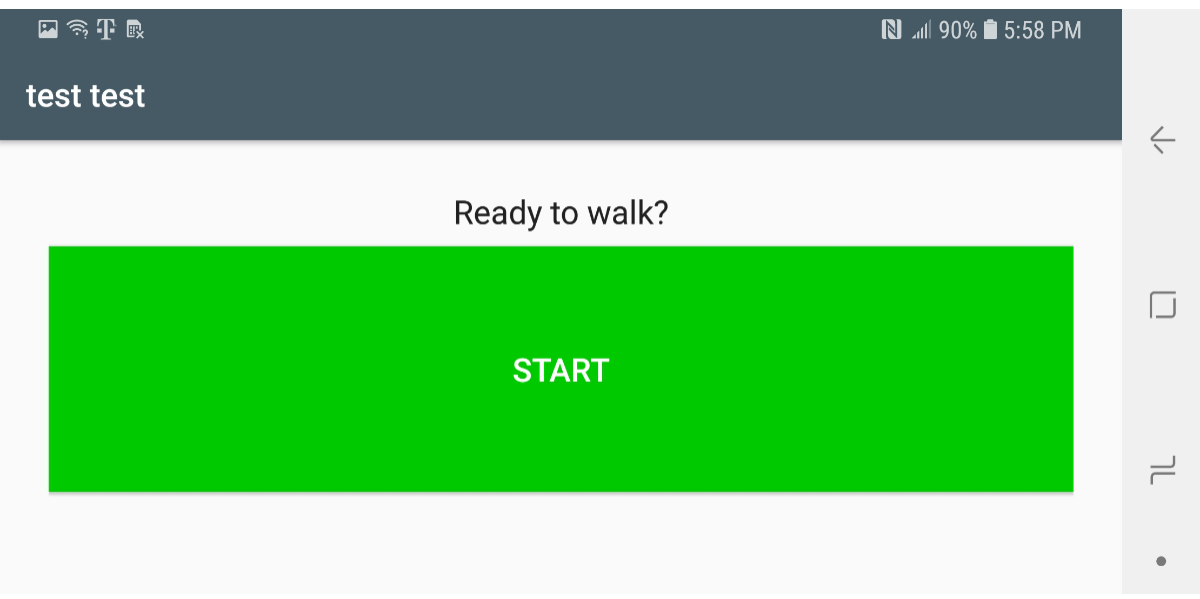
Start fragment of the mobile health app.

**Figure 3:**
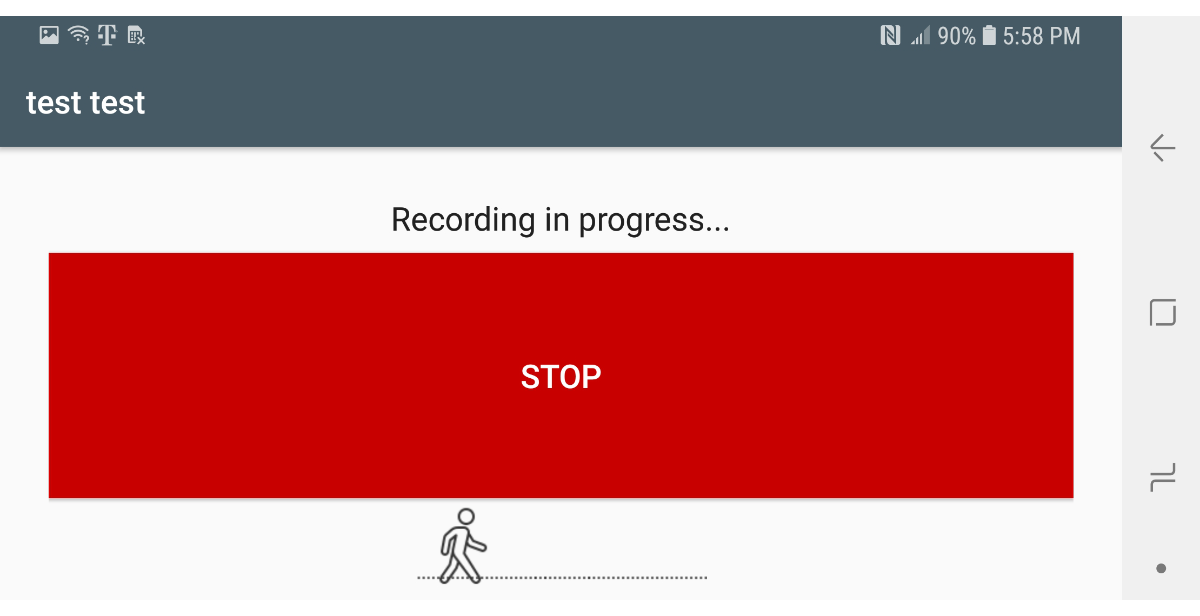
Stop fragment of the mobile health app.

**Figure 4:**
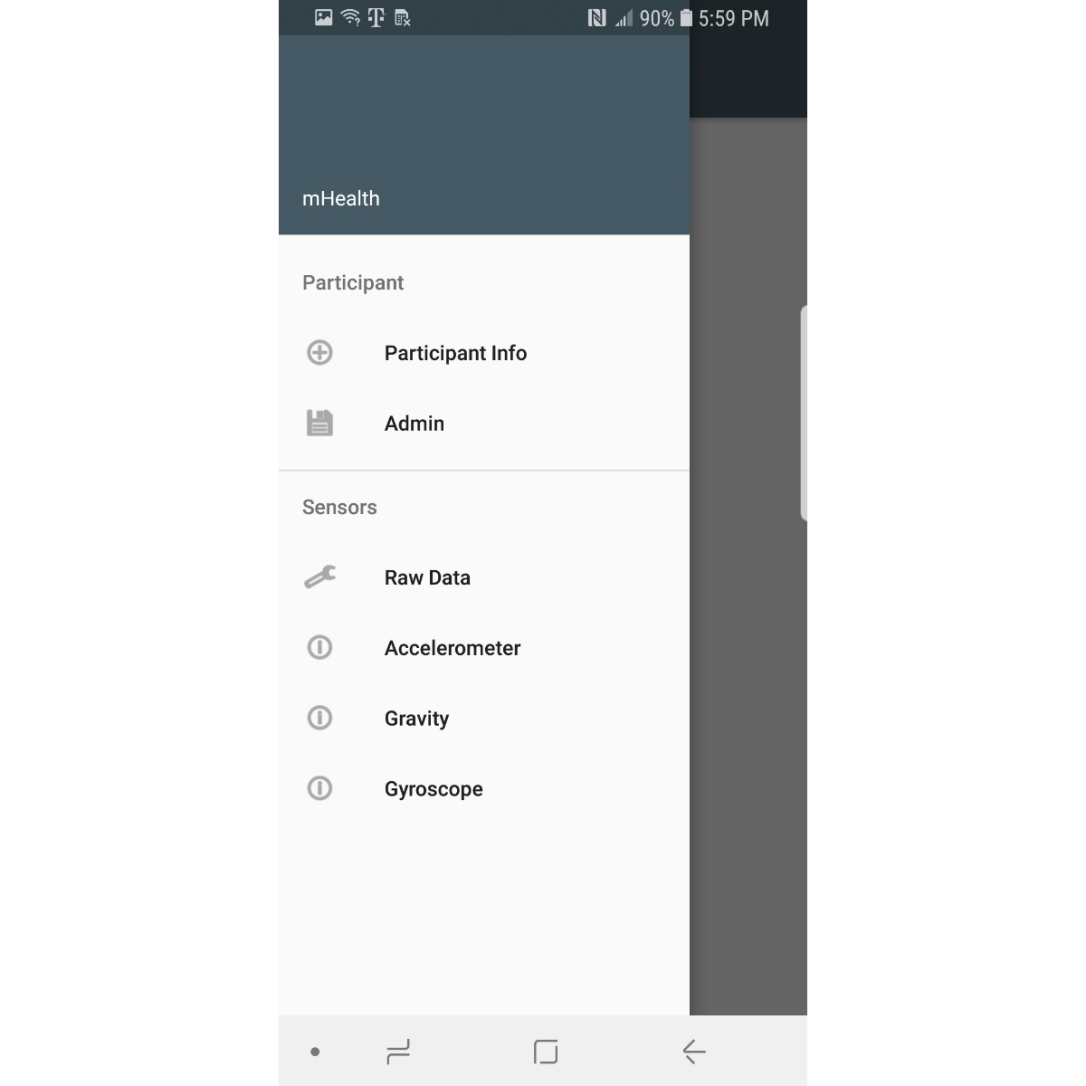
Menu widget.

**Figure 5:**
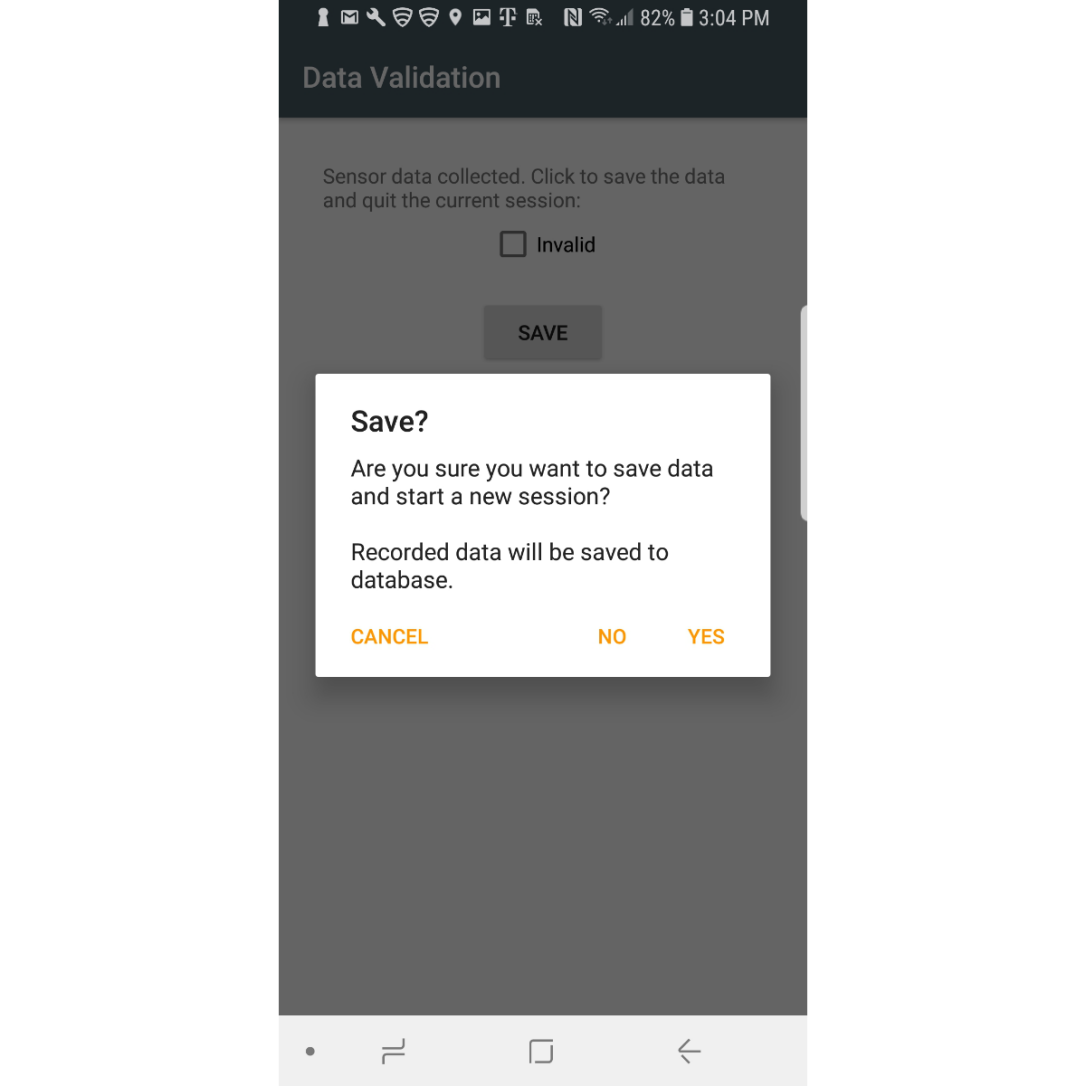
Confirm walk test.

**Figure 6:**
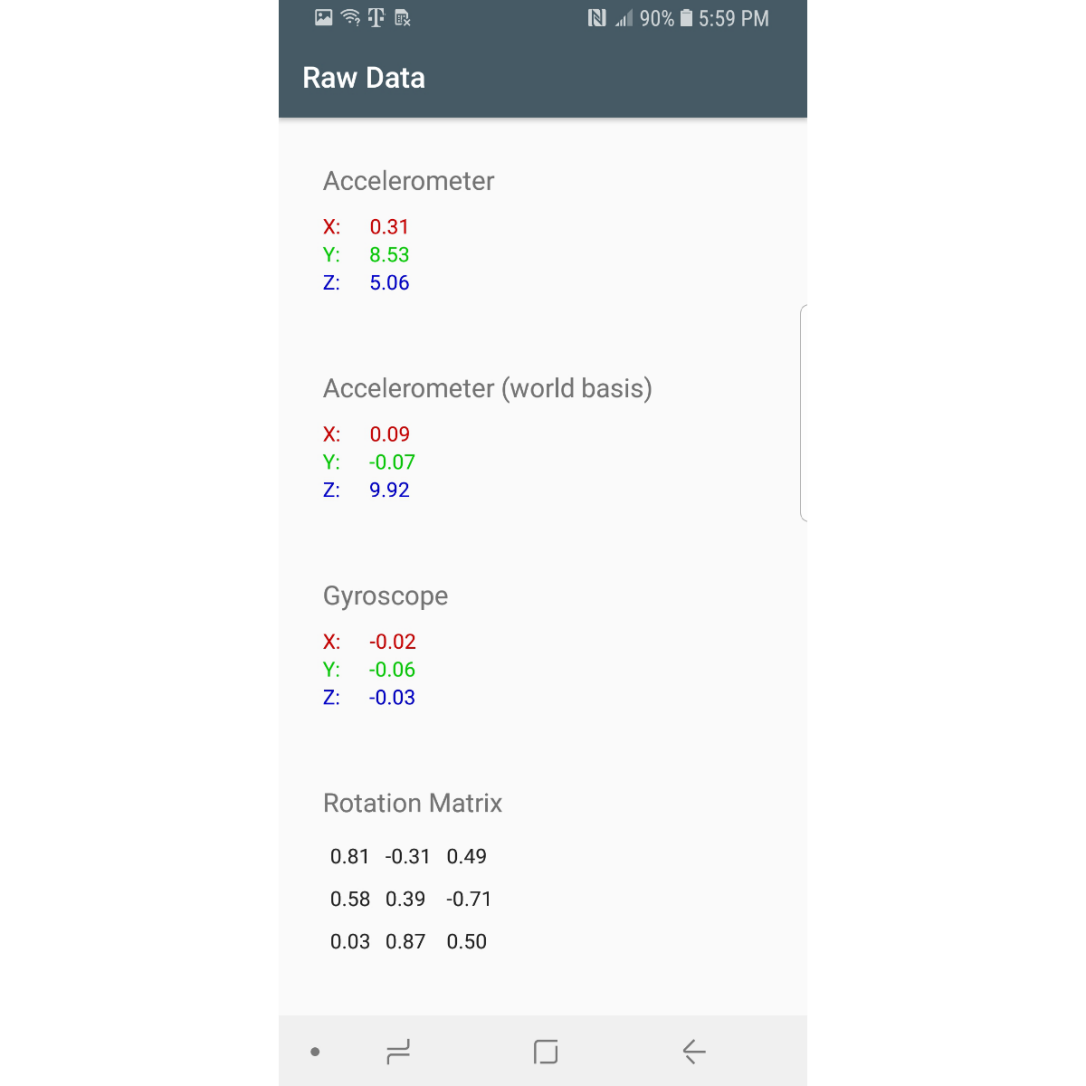
Sensor data matrix.

**Figure 7:**
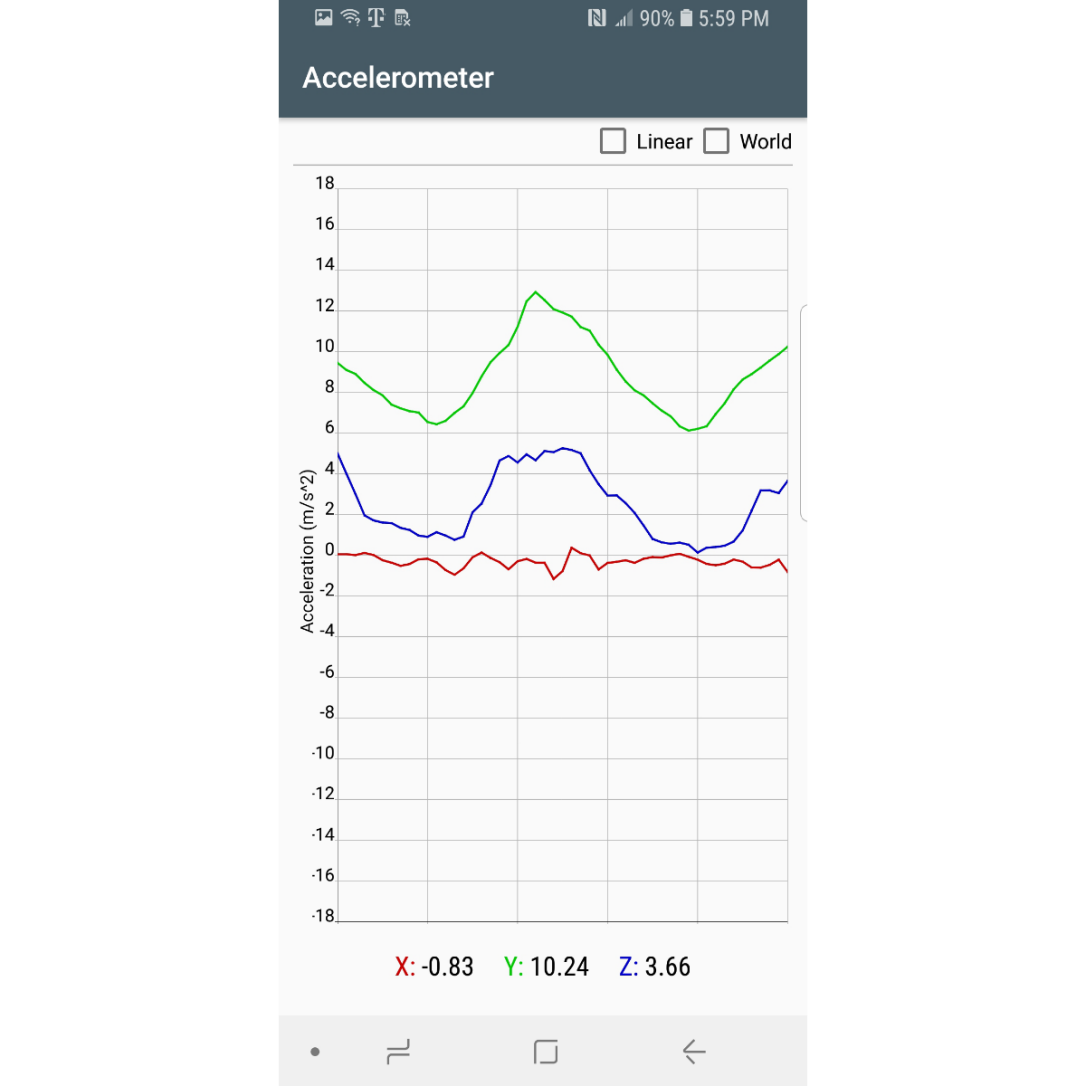
Sensor data graph.

The mHealth app was developed to measure and record rotational data in real time by using an Android smartphone’s motion sensors. Figures 1-7 show the mHealth app in use to gather the sensor data. The app was programmed for Android mobile platforms with software development kits greater than 21 using Android Studio [19]. This research used the app on the default settings of the Samsung Galaxy S8 [20] with Android 7.0 mobile operating system. The code is written in Java using the Android application programming interface [21]. This app uses the Android sensor framework to access sensor data as part of the hardware package that consists of 3 classes and 1 interface. The classes are SensorManager, Sensor, and SensorEvent. SensorManager accesses the device’s sensors, Sensor obtains the list of available sensors, and SensorEvent creates the sensor object that includes the raw sensor data. The interface, SensorEventListener, receives notifications when a sensor value or accuracy changes [22].

Sensors used in this app include the gyroscope, accelerometer, gravity, and magnetic sensors. The gravity and magnetic sensors are used to calculate the rotation matrix using the getRotationMatrix method, which belongs to the SensorManager class [23]. Raw sensor data visualizations are created using the Androidplot library [24]. The collected sensor data are stored in an SQLite database [25]. Two comma-separated value files are created from the saved participant information and sensor values. These files are saved on the device for further data analysis. Figure 1 shows the main fragment in the mHealth app. In the main fragment, the mHealth obtains the participant’s status such as any pain (back, leg, head, etc), any medication in the last 3 days, any problems walking, concussion experience, gender, race/ethnicity, height, and weight. After touching the “SUBMIT” button in the main fragment, the mHealth app proceeds to the fragment shown in Figure 2. After selecting the “START” button (Figure 2), mHealth changes the fragment to Stop Fragment, as shown in Figure 3. Then, the participant walks straight forward for 2-3 minutes and then returns to the starting location. After the participant returns to the starting location, the participant touches the “STOP” button, as shown in Figure 3. The walking data are recorded between the time of selecting the “START” button (Figure 2) and the time of selecting the “STOP” button (Figure 3). After touching the “STOP” button, an administrator opens the widget in the mHealth app (Figure 4) to save the data by swiping from left to right on the smartphone screen. The “Admin” button (Figure 4) is used to save the recorded walking data. When selecting the “Admin” option (Figure 4), the mHealth app moves to the Save Fragment. There is 1 checkbox (Figure 5) to confirm whether the walking data are valid or not. The checkbox is for indicating whether the test was invalidated by an interruption or an unexpected event during recording. By selecting the “SAVE” button (Figure 4), the data are saved to an SQLite database and a comma-separated value file on the local disk. The mHealth app has several functions to analyze the raw data in real time. By selecting functions such as “Metrics,” “Accelerometer,” and “Gyroscope” (Figure 4), the mHealth app displays the current status of the raw data, as shown in Figure 6 and Figure 7. Figure 6 shows the current accelerometer, gyroscope, and rotation matrix in real time. Figure 7 shows the graph of the sensor data.

### Data Collection

**Figure 8:**
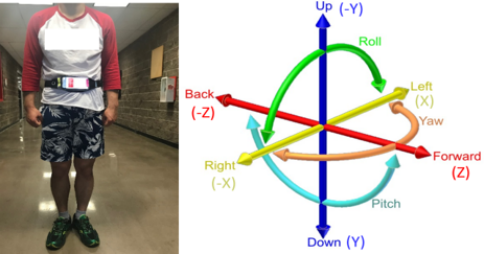
XYZ-axis orientations of the smartphone during the walking balance test. X-axis is the right (−) and left (+) motions of the participant. Y-axis is the up (−) and down (+) motions of the participant. Z-axis is the forward (+) and backward (−) motions of the participant.

The mHealth app was tested with 3 participants. Two participants were healthy (1 female and 1 male) and they did not have any problems walking; these 2 participants were in their twenties. The third participant with back pain was in his thirties and he had little pain in his back; however, he had no problem walking and we could not find any difference in his walking compared to the other participants. During the experiment, all the participants wore the smartphone in a pocket of a waistband at the center of their body, as shown in Figure 8. The smartphone was placed on the waistline with its top frontside facing the right side of the body. The participants were instructed to walk straight at their own pace in an indoor hallway of a building. They walked a distance of about 400 feet. They walked for about 2-3 minutes in a straight line and then returned to the starting location. The duration of the test time for each participant was 4-5 minutes. They were allowed only a single trial for the walking movement test.

The smartphone stored the motion sensor data in the real-time mHealth app on the phone. The app collected the sensor data in 20 milliseconds. The sensor data such as accelerometer (x, y, and z), gyroscope (x, y, and z), and rotation matrix (3×3) were stored in an SQLite database and a comma-separated value file. Data corresponding to the first 10 seconds and the last 10 seconds of each participant session were removed to account for the press time of the start and stop button activation within the mHealth app. Among the sensor data, the rotation matrix was used to determine the walking balance. Because the rotation matrices can represent the rotation of the origin frame into the reference frame, the rotation matrix is commonly used to measure posture balance [26-31]. The next section explains the analysis method using the rotation matrix.

### Data Analysis

Considering the differentiation in the balance control of the participants while walking, the rotation vector data are the most effective [32,33]. Therefore, the rotation vector was extrapolated from the rotation matrix data recorded with the smartphone. In the mHealth app, the xyz-axis rotation vectors of the middle of the waistline were identified as the center of gravity of each participant. The x-axis represents the body motion angle between the right and left side movements of a participant. The y-axis represents the body motion angle between the up and down movements of a participant. The z-axis represents the body motion angle between the forward and backward movements of a participant (Figure 8). The xyz-axis rotation vector was obtained from the rotation matrix. A rotation matrix is a matrix that is used to perform a rotation in Euclidean space. The Euler’s theorem on the axis of a three-dimensional rotation is formulated as follows:

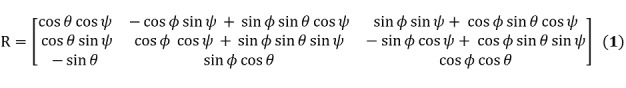



If R is a 3×3 orthogonal matrix (R^T^R = RR^T^ = I) and R is proper (det(R) = +1), then there is a nonzero vector “v” satisfying Rv = v [34]. The rotation matrix data were collected using the rotation sensor in the Android Open Source Project. Using the rotation sensor, the mHealth app determined the rotation matrix [34] like equation 1. The rotation matrix was as follows:

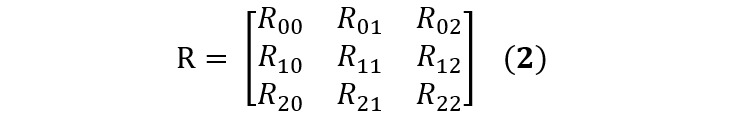



From the equation 2 rotation matrix (3×3), we extracted the rotation vector x-axis (R_X_), y-axis (R_Y_), and z-axis (R_Z_) by using the following formula [35]:

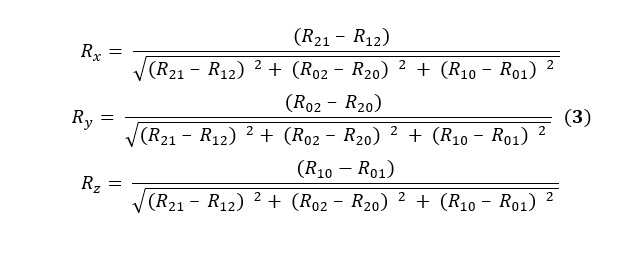



Although each participant wore the smartphone at the same location of his/her body, the sensors in the smartphone appeared to be located with slightly different slopes. Therefore, we used the difference of rotation between current time (t) and previous time (t-1) for the data analysis:

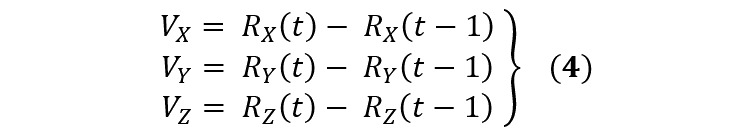



**Figure 9:**
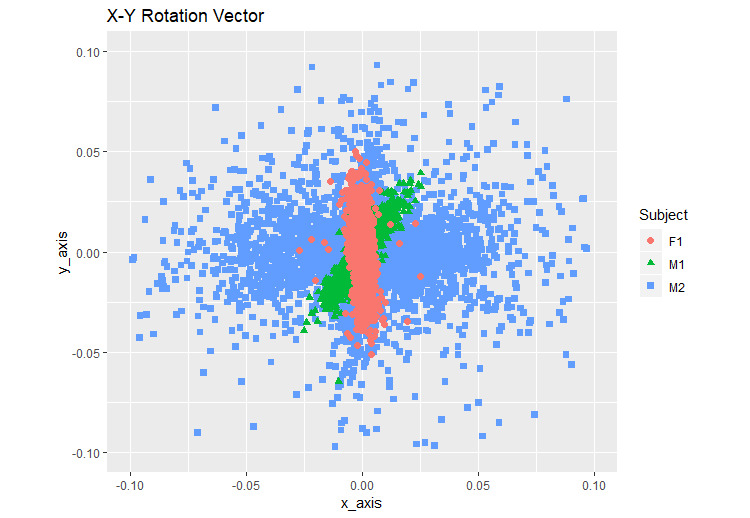
X-Y rotation vector. F1: healthy female; M1: healthy male; M2: male with back pain.

**Figure 10:**
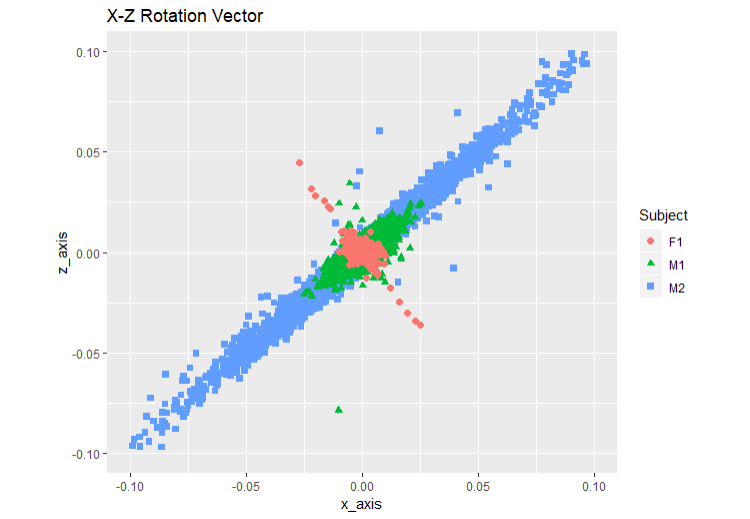
X-Z rotation vector. F1: healthy female; M1: healthy male; M2: male with back pain.

**Figure 11:**
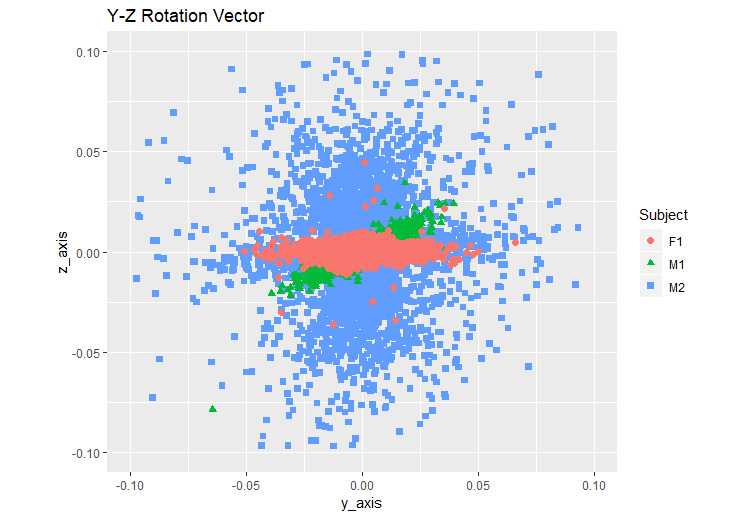
Y-Z rotation vector. F1: healthy female; M1: healthy male; M2: male with back pain.

For comparison across the participants, the difference in the rotation vector values was plotted as 2D graphs using ggplot2 in R (Figures 9-11).

## Results

We collected constant motion data by using the sensors in the smartphone. Thereafter, to understand constant motion changes in the middle of the participants’ waistline (umbilicus) as the center of gravity, the rotation vector values of the 3 participants were extracted and compared. There were no differences in the rotation matrix data of each participant. However, we found a difference among the participants when applying the formula (equation 4) into the data.

We calculated the variation in each step of the participants by using the formula (equation 4) (Table 1). Figures 9-11 show the illustrative rotation data, which were obtained using the formula (equation 4) by 2 combined axes during the same time period recorded by the smartphones from 2 healthy individuals and 1 individual with back pain. Illustrative rotation x-axis data show the right (−) to left (+) body motions on the x-axis. Illustrative rotation y-axis data show the up (−) to down (+) body motions on the y-axis. Illustrative rotation z-axis data recorded the forward (+) to backward (−) body motions on the z-axis. Healthy participants appeared to have narrower ranges of right-to-left and up-and-down motions than the participant with back pain who had the widest range of motion while walking. The results showed that there were differences in the body xyz-axis variations of rotation vector data between the participant with back pain and the healthy participants. The variation in the y-axis rotation of the participant with back pain was slightly wider than that of the healthy participants. Moreover, the participant with back pain appeared to have more variations in xz-axis rotations than the healthy participants, suggesting that he had a wider range of motion between right and left and forward and backward movements while walking compared to the healthy participants (Figures 9-11).

**Table 1:** Statistical information of the variations in the rotation vector data.

Participant, rotation vector		Maximum	Minimum	Mean	Variance	SD
**Healthy male participant**						
	x-axis	0.025	–0.025	0	0	0.004
	y-axis	0.039	–0.065	0	0	0.006
	z-axis	0.034	–0.079	0	0	0.004
**Healthy female participant**						
	x-axis	1.715	–1.715	0	0.085	0.292
	y-axis	0.202	–0.21	0	0	0.017
	z-axis	1.184	–1.489	0	0.036	0.188
**Male participant with back pain**						
	x-axis	1.467	–1.466	0	0.017	0.129
	y-axis	0.944	–0.534	0	0	0.021
	z-axis	1.492	–1.494	0	0.017	0.13

## Discussion

### Principal Results

In this study, we developed an mHealth app to measure the body movements of 3 participants wearing the smartphone on their waists while walking. The motion sensors of the smartphone were used to measure the walking movements and to analyze the rotation matrix data with the proposed method, which shows the variables of walking over time. The difference in each walking step of the 3 participants was compared using a formula (equation 4). Table 1 shows the statistical information of the walking movement data after applying the formula. The healthy male participant had the smallest standard deviation (Table 1), indicating that he had the most balanced walking movement. The healthy female participant showed symmetrical walking movement in the left-to-right (x-axis) and up-and-down (y-axis) movements in the variations of the x-y axis rotation vector (Figure 9), indicating that she had lesser bias in gait than the other two participants. The x-axis standard deviation in the healthy female participant was the highest because female hip is bigger than male hip [36]. The standard deviation of y-axis and z-axis of the participant with back pain was the highest (Table 1), indicating that he had a wider movement than the other two healthy participants. Thus, the participant with back pain had a lesser balanced walking movement while walking than the other participants. Our app showed that the participant with little back pain has a wide range of movement during walking. Specifically, this participant appeared to show more variations in the right-to-left and forward-to-backward movements.

### Limitations

This study has several limitations. The mHealth app was tested only in 3 participants. Nevertheless, the proposed mHealth app can effectively capture differences in the postural control during walking between healthy individuals and individuals with back pain. Further, our mHealth app is not available currently for observing the data in real time while walking. Therefore, we will implement a program that will make it possible to observe the data while walking.

### Future Directions

In future research, we will conduct feasibility and efficacy testing with large pools of individuals with reduced physical mobility. For the tests, we are planning to collect walking data from high school students because we believe that this younger group is ideal for researching on walking data. This younger sample is lesser affected by aging/diseases related to walking than an older group. Further, high school students will be in school for 2 or 3 years before graduation, which is a long period for tracking their walking movements. After we collect enough walking movement data, we will compare our findings with the results from previous mHealth studies. In addition, any variable that may affect postural balance during walking such as body habitus (ie, slim vs obese) or level of daily physical activities will be considered in data collection and analysis with various sensors in smartphones. We will also consider evaluating the impact of the app at the health system level by using outcomes such as health care utilization and medication use. This study is beneficial since it provides a useful method for medical evaluation in rehabilitation and physical fitness and a means for participants to maintain a state of well-being. This research can be used to classify walking movements between people who have walking-related diseases and normal people. This classification can help in diagnosing their diseases. Thereafter, we will research the classification of walking movements based on diseases that affect walking.

### Conclusions

Our study shows that mHealth and the walking rotation vector can be used to define the body walking movements. Currently, the most widely used assessments for measuring postural control are laboratory-based such as the previous walking movement research [12,13] and human balance research [2,3]. Along with requiring specialized equipment, such assessments typically do not provide real-time feedback. However, our proposed mHealth app and analysis methods support home-based measurements. The findings of this study support our mHealth app as a low-cost and easy-to-use alternative with minimal equipment required that provides sensitive walking balance assessment [6].

## Abbreviations

mHealth: mobile health
